# Predictive model for epileptogenic tubers from all tubers in patients with tuberous sclerosis complex based on ^18^F-FDG PET: an 8-year single-centre study

**DOI:** 10.1186/s12916-023-03121-0

**Published:** 2023-12-18

**Authors:** Zhongke Wang, Yang Li, Zeng He, Shujing Li, Kaixuan Huang, Xianjun Shi, Xiaoqin Sun, Ruotong Ruan, Chun Cui, Ruodan Wang, Li Wang, Shengqing Lv, Chunqing Zhang, Zhonghong Liu, Hui Yang, Xiaolin Yang, Shiyong Liu

**Affiliations:** 1 Department of Neurosurgery, Armed Police Hospital of Chongqing, Chongqing, China; 2 Department of Neurosurgery, Comprehensive Epilepsy Center, Xinqiao Hospital, Army Medical University, Chongqing, China; 3 Department of Virology, State Key Laboratory of Pathogen and Biosecurity, Beijing Institute of Microbiology and Epidemiology, Beijing, China; 4 Department of Radiology, Xinqiao Hospital, Army Medical University, Chongqing, China; 5 Department of Neurology, Xinqiao Hospital, Army Medical University, Chongqing, China; 6 Chongqing Institute for Brain and Intelligence, Guangyang Bay Laboratory, Chongqing, China

**Keywords:** Tuberous sclerosis complex, Epileptogenic tubers, Epilepsy, ^18^F-FDG PET, Machine learning

## Abstract

**Background:**

More than half of patients with tuberous sclerosis complex (TSC) suffer from drug-resistant epilepsy (DRE), and resection surgery is the most effective way to control intractable epilepsy. Precise preoperative localization of epileptogenic tubers among all cortical tubers determines the surgical outcomes and patient prognosis. Models for preoperatively predicting epileptogenic tubers using ^18^F-FDG PET images are still lacking, however. We developed noninvasive predictive models for clinicians to predict the epileptogenic tubers and the outcome (seizure freedom or no seizure freedom) of cortical tubers based on ^18^F-FDG PET images.

**Methods:**

Forty-three consecutive TSC patients with DRE were enrolled, and 235 cortical tubers were selected as the training set. Quantitative indices of cortical tubers on ^18^F-FDG PET were extracted, and logistic regression analysis was performed to select those with the most important predictive capacity. Machine learning models, including logistic regression (LR), linear discriminant analysis (LDA), and artificial neural network (ANN) models, were established based on the selected predictive indices to identify epileptogenic tubers from multiple cortical tubers. A discriminating nomogram was constructed and found to be clinically practical according to decision curve analysis (DCA) and clinical impact curve (CIC). Furthermore, testing sets were created based on new PET images of 32 tubers from 7 patients, and follow-up outcome data from the cortical tubers were collected 1, 3, and 5 years after the operation to verify the reliability of the predictive model. The predictive performance was determined by using receiver operating characteristic (ROC) analysis.

**Results:**

PET quantitative indices including SUVmean, SUVmax, volume, total lesion glycolysis (TLG), third quartile, upper adjacent and standard added metabolism activity (SAM) were associated with the epileptogenic tubers. The SUVmean, SUVmax, volume and TLG values were different between epileptogenic and non-epileptogenic tubers and were associated with the clinical characteristics of epileptogenic tubers. The LR model achieved the better performance in predicting epileptogenic tubers (AUC = 0.7706; 95% CI 0.70–0.83) than the LDA (AUC = 0.7506; 95% CI 0.68–0.82) and ANN models (AUC = 0.7425; 95% CI 0.67–0.82) and also demonstrated good calibration (Hosmer‒Lemeshow goodness-of-fit *p* value = 0.7). In addition, DCA and CIC confirmed the clinical utility of the nomogram constructed to predict epileptogenic tubers based on quantitative indices. Intriguingly, the LR model exhibited good performance in predicting epileptogenic tubers in the testing set (AUC = 0.8502; 95% CI 0.71–0.99) and the long-term outcomes of cortical tubers (1-year outcomes: AUC = 0.7805, 95% CI 0.71–0.85; 3-year outcomes: AUC = 0.8066, 95% CI 0.74–0.87; 5-year outcomes: AUC = 0.8172, 95% CI 0.75–0.87).

**Conclusions:**

The ^18^F-FDG PET image-based LR model can be used to noninvasively identify epileptogenic tubers and predict the long-term outcomes of cortical tubers in TSC patients.

**Supplementary Information:**

The online version contains supplementary material available at 10.1186/s12916-023-03121-0.

## Background

Tuberous sclerosis complex (TSC) is a rare autosomal dominant genetic syndrome with an incidence of 1 in 6000–22000 live births. Approximately 85% of TSC patients have TSC1 or TSC2 gene mutations, which cause overactivation of the mTOR signalling pathway. TSC is a typical cause of neurodevelopmental delay, and over 54% of TSC patients suffer from drug-resistant epilepsy (DRE) [1–3]. Our previous nationwide multicentre retrospective study reported the China expert consensus on the surgical treatment of TSC-related intractable epilepsy and showed that surgery is the most effective way to control intractable epilepsy in patients with TSC [4, 5]. Multiple cortical tubers are typical manifestations in patients with TSC, and a previous study showed that electrographic seizures originated from the cortical tubers and perituberal cortex [6]. Therefore, precise preoperative identification of epileptogenic tubers from all cortical tubers is the key to determining the resection approach, surgical outcomes, and prognosis for TSC-related epilepsy [7–9]. Epileptogenic tubers can be identified by comprehensive preoperative assessments, including neurological physical examinations, genetic tests, computed tomography (CT), magnetic resonance imaging (MRI), ^18^F-fluorodeoxyglucose positron emission tomography/computed tomography (^18^F-FDG PET/CT), scalp video-EEG and intracranial EEG monitoring [10]. Epileptic discharges on intracranial EEG monitoring are reliable signals for localizing epileptogenic tubers, but this procedure is invasive. Given growing evidence of the potential for imaging biomarkers to predict epileptogenic foci, noninvasive neuroimaging monitoring has become of increasing interest in identifying epileptogenic tubers in TSC patients.

The safety and efficacy of different imaging modalities for locating epileptogenic foci varies widely. PET is considered an essential noninvasive method for preoperative evaluation in epilepsy patients, as it can safely reflect dynamic, seizure-related metabolism, especially when MRI and EEG monitoring are discordant or inconclusive [11, 12]. And machine learning based on ^18^F-FDG PET images can be used to accurately lateralize and classify MRI-negative temporal lobe epilepsy [13]. Although ^18^F-FDG PET has become an indispensable part of preoperative evaluations for TSC patients with intractable epilepsy, the precise localization of epileptogenic tubers based on ^18^F-FDG PET images has not been fully evaluated due to a lack of systematic studies.

Quantitative ^18^F-FDG PET analysis has been used to explore the distribution of hypometabolism in patients with focal epilepsy in different brain regions, as the resulting quantitative indices are an informative tool for identifying potential epileptogenic zones [14, 15]. Positive correlations have been identified between the maximum standard uptake value (SUV) and the frequency of lateralized periodic discharges in patients with intractable epilepsy [16], and quantitative ^18^F-FDG PET asymmetry features predict long-term seizure recurrence in refractory epilepsy. Our previous research reported the SUVs could predict the localization of epileptogenic foci in patients with focal cortical dysplasia type IIb and TSC [17]. According to these results, we hypothesize the quantitative indices of ^18^F-FDG PET would provide the potential value in identifying epileptogenic tubers in patients with TSC.

In this study, we constructed a predictive model based on ^18^F-FDG PET images to accurately identify epileptogenic tubers from multiple cortical tubers before surgery, predict the long-term outcomes of epileptogenic tubers, and provide guidance to clinicians on precise surgical intervention in TSC.

## Methods

### Study design and cohorts

**Fig. 1:**
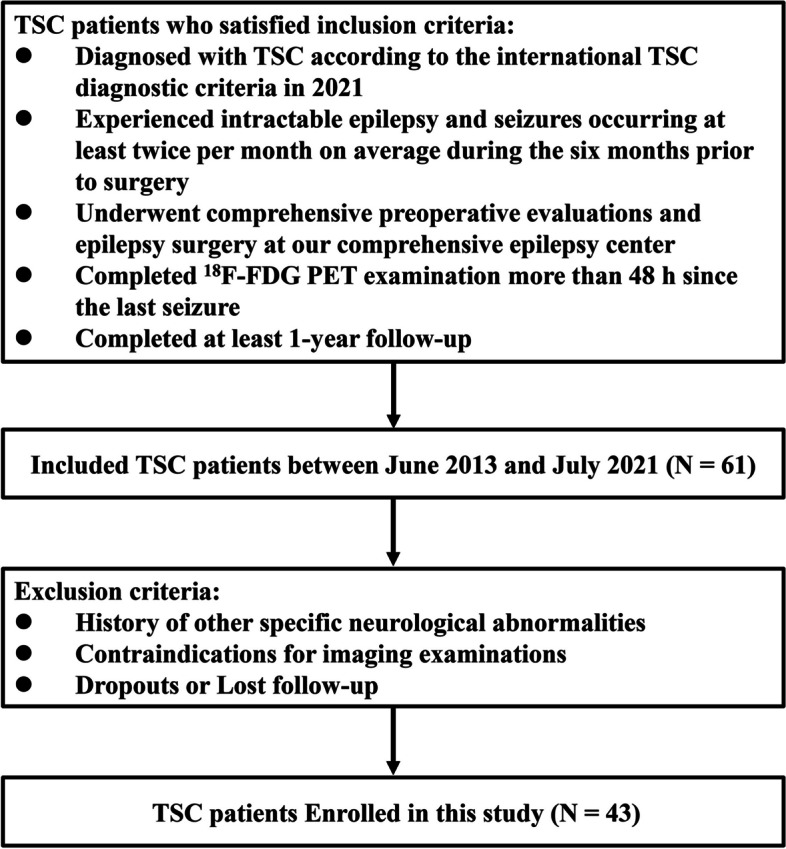
Flow diagram. Flowchart shows the patient inclusion and exclusion criteria

This study was approved by our institutional review board, and all subjects signed informed consent forms. We selected consecutive TSC patients who presented to the comprehensive epilepsy center from June 2013 to July 2021. The inclusion criteria were (i) patients diagnosed with TSC according to the diagnostic criteria in 2021 [3]; (ii) intractable epilepsy and seizures occurring at least twice per month on average during the 6 months prior to surgery; (iii) comprehensive preoperative evaluations and epilepsy surgery at our comprehensive epilepsy centre; (iv) ^18^F-FDG PET examination completed more than 48 h since the last seizure; and (v) at least 1 year of follow-up. The exclusion criteria were (i) patients with other specific neurological abnormalities, mainly including encephalitis, hydrocephalus, intracerebral haemorrhage or cerebrovascular disease; (ii) contraindications for imaging examinations; and (iii) who dropped out of the study or loss to follow-up. The study flowchart is presented in Fig. 1.

Clinical characteristics (including data on sex, age at onset, frequency of seizures, number of antiseizure medications [ASMs], and number of tubers) were collected. Comprehensive preoperative examinations, including neurological physical examinations, genetic tests, MRI, ^18^F-FDG PET/CT, scalp video-EEG and stereoelectroencephalography (SEEG), were performed by neurosurgeons, neurologists and neuroradiologists to determine whether and how to perform epilepsy surgery on the TSC patients. We first chose a noninvasive method to identify the epileptogenic tubers based on our and other previous studies [4, 18, 19]. An epileptogenic tuber was defined as (i) a cortical tuber with focal ictal and interictal epileptiform discharges in the same brain region on scalp EEG with consistent seizure symptoms; (ii) an outstanding cortical tuber with focal ictal or interictal epileptiform discharges in the same brain region on scalp EEG with consistent seizure symptoms; or (iii) an outstanding cortical tuber with focal ictal and interictal epileptiform discharges in the same brain region on scalp EEG. Outstanding cortical tubers described were defined as having a size > 3–4 cm and containing a nidus of calcification according to imaging results. For those patients with multiple epileptogenic cortical tubers or discrepancies among clinical symptoms, imaging results and electrophysiology findings, intracranial electrodes were invasively implanted to record electrical activity and identify epileptogenic tubers. In addition, resected “non-epileptogenic tubers” in this study are adjacent to epileptogenic tubers, exposed to the same surgical field with the epileptogenic tubers and located in the non-functional areas, and these tubers are large, prominent or calcified tubers but without epileptic discharges. These tubers have the potential to become epileptogenic tubers, strongly suggest pharmacoresistant epilepsy in TSC, influence postoperative seizure freedom and should be considered good indicators for epilepsy surgery [4, 20]. Patients finished their last follow-up in July 2022. The clinical characteristics of the patients are shown in Table 1.

**Table 1:** Clinical characteristics of patients with TSC (*n* = 43)

Characteristics		Values
**Female/male**		20(46.5)/23(53.5)
**Type of variation**		
**TSC1**		8(18.6)
**TSC2**		29(67.4)
**No variation**		6(14)
**Age at onset (month)**		20.16(1–156)
**Frequency of seizure**		
**Per day**		26(60.5)
**Per week**		10(23.2)
**At least twice per month**		7(16.3)
**Number of AEDs**		2.19(1–4)
**Number of tubers**		
**1–3**		8(18.6)
**4–6**		26(60.5)
** > 6**		9(20.9)
**Ictal scalp EEG**		
**Focal**		10(23.2)
**Lateralized**		12(27.9)
**Multifocal**		14(32.6)
**Generalized**		7(16.3)
**Intracranial EEG**		
**Subdural electrode EEG**		16(37.2)
**Stereo-EEG**		13(30.2)
**No intracranial EEG**		14(32.6)
**Surgery approach**		
**(Multiple) tuberectomy**		17(39.5)
**(Multiple)lobectomy**		11(25.6)
**Lobectomy + tuberectomy**		15(34.9)
**Post-surgical seizure outcomes**		
**1 year follow**^**a**^	**SF**	32(74.4)
	**No-SF**	11(25.6)
**3 years follow**^**b**^	**SF**	26(68.4)
	**No-SF**	12(31.6)
** > 5 years follow**^**c**^	**SF**	21(65.6)
	**No-SF**	11(34.4)

*Abbreviations*: *TSC* tuberous sclerosis complex, *AED* antiepileptic drug, *SF* seizure freedom

Values are *n* (%) or mean (range)

^a^Available patient data *n* = 43

^b^Available patient data *n* = 29

^c^Available patient data *n* = 13

### Imaging data collection and processing

The criteria are summarized as follows: at least 4 h of fasting before FDG PET/CT, blood glucose level at the time of FDG injection < 11.1 mmol/L, and PET acquisition was done 1 h after the injection of 3 MBq/kg of FDG. In addition, MRI scans were performed with a 3.0-T system with a vendor-produced 32-channel head coil. Patients were in an awake, resting state, and no patients had seizures during the imaging scans. Image registration was utilized to spatially align the ^18^F-FDG PET and T2Flair MRI images using CT images as a reference, and the registered image showed the spatial correspondence between PET and MRI images (Additional file 1: Fig. S1, Step 1–2). Then, regions of interests (ROIs) were delineated on the T2Flair MRI images and the delineated ROIs also applied to the PET images (Additional file 1: Fig. S1, Step 3). The quantitative indices of the delineated ROIs were further extracted based on the PET images (Additional file 1: Fig. S1, Step 4). In each delineated ROI, 19 quantitative indices that reflect the clinical significance of the PET image were identified [17, 21–24]. A detailed explanation of these indices is shown in Additional file 1: Tab. S1. Researchers blinded to the clinical and imaging features delineated the ROIs and analysed the imaging data independently. All processing procedures were performed under the guidance of experienced PET/CT specialists.

### Model development and evaluation

Logistic regression (LR), linear discriminant analysis (LDA) and artificial neural network (ANN) models were constructed using R 4.2.1 software (R Foundation for Statistical Computing, Vienna, Austria). ROC analysis was used to confirm the identification performance of all models. The relationship between the PET quantitative indices and the probability of identifying an epileptogenic tuber was visualized at the bottom of the nomogram. Calibration curves were used to assess the agreement between the predicted probability and the actual proportion, and the Hosmer–Lemeshow test was used to estimate the calibration of the nomogram model. In addition, decision curve analysis (DCA) and clinical impact curve (CIC) were performed to evaluate the utility of the nomogram.

### Western blot

The proteins of the resected brain tissues from TSC patients were extracted, and their concentration was measured using a BCA protein assay. The extracted protein was mixed with 5 × loading buffer, electrophoresed in 10% sodium dodecyl sulfate polyacrylamide gel and transferred onto polyvinylidene difluoride membranes. The membranes were then blocked in 5% skim milk for 2 h at room temperature. Then, the membranes were incubated with the primary antibodies overnight at 4 °C, including anti-mTOR (1:1000, Abcam) and anti-GAPDH (1:1000, Abcam). After washing, the membranes were incubated with anti-rabbit or anti-mouse secondary antibodies for 1 h at room temperature. Specific protein bands were visualized in the membranes using the chemiluminescence method, and the optical density of the protein bands was analysed in Image-Pro Plus software.

### Statistics

Continuous variables were compared by the unpaired two-tailed *T* tests. The relationship between quantitative indices and epileptogenesis was evaluated using logistic regression analysis. Bivariate correlation analyses were carried out using Spearman’s rank correlation test. Receiver operating characteristic curve (ROC) analysis was performed to evaluate the performance of the models. The area under the curve (AUC) was calculated to illustrate the diagnostic power of the models. A nomogram was built to help guide clinicians, and DCA was performed to evaluate the utility of the nomogram. R 4.2.1 software was used for data analysis. Significance was set at *P* < 0.05.

## Results

### Patient clinical characteristics

Data from 61 TSC patients who underwent epilepsy surgery in our centre were collected. Eighteen patients were excluded based on the exclusion criteria. Forty-three patients (20 female, 23 male) were ultimately enrolled in this study (Fig. 1).

The clinical characteristics of the TSC patients were collected (Table 1). According to gene testing, 8 patients had a TSC1 variant, 29 had a TSC2 variant and 6 patients had no gene variants. The average age at onset was 20.16 months (range 1–156 months). Seizures occurred every day in 26 patients, per week in 10 patients and at least twice per month in 7 patients. The average number of ASMs was 2.19 (range 1–4). Eight patients had 1–3, twenty-six patients had 4–6 and nine patients had more than 6 cortical tubers. According to the ictal scalp EEG findings, 10, 12, 14 and 7 patients had focal, lateralized, multifocal and generalized epileptic discharges, respectively. Furthermore, 16 patients underwent subdural EEG monitoring, and 13 patients underwent SEEG monitoring. Tuberectomy or multiple tuberectomy was performed on 17 patients, lobectomy or multiple lobectomy was performed on 11 patients, and lobectomy plus tuberectomy was performed on 15 patients. One-year follow-up was performed for all patients, and 32 patients were seizure-free. Three-year follow-up was performed for 38 patients, and 26 patients were seizure-free. An over-5-year follow-up was performed for 32 patients, and 21 patients were seizure-free.

### Correlation between quantitative indices and epileptogenic tubers

Overall, 235 tubers (71 epileptogenic and 164 non-epileptogenic) from 43 patients were identified, and their PET quantitative indices were extracted and analysed. The results showed that the SUVmean, SUVmax, volume, total lesion glycolysis (TLG), third quartile, upper adjacent and standardized added metabolic activity (SAM) were associated with epileptogenic tubers (Table 2), and the SUVmean, SUVmax, volume, TLG and root-mean-square deviations (RMSD) values were different between epileptogenic tubers and non-epileptogenic tubers (Additional file 1: Tab. S2). Based on these results, we further selected the SUVmean, SUVmax, volume and TLG indices to investigate the association of PET quantitative indices and epileptic characteristics in TSC patients with a single epileptogenic tuber and found that SUVmean, SUVmax and TLG were negatively associated with seizure frequency and duration (Additional file 1: Fig. S2). Abnormal activation of the mTOR pathway is the pathogenic mechanism of epilepsy in TSC [25]. Intriguingly, SUVmean and SUVmax were negatively associated with activated mTOR expression in resected epileptogenic tubers (Additional file 1: Fig. S3). The above results indicated that the PET quantitative indices have potential predictive value for epileptogenic tubers.

**Table 2:** Relationship between quantitative indices and epileptogenic tubers in TSC patients

Quantitative indices	β	SE	Wald	*P* value	Exp(β)	95.0% CI	
**SUVmean**	** − 1.101**	**0.471**	**5.474**	0.019*	**0.332**	**0.132**	**0.836**
**SUVmax**	** − 1.472**	**0.528**	**7.768**	0.005**	**0.229**	**0.082**	**0.646**
**Volume**	**0.911**	**0.330**	**7.608**	0.006**	**2.487**	**1.302**	**4.750**
**TLG**	** − 0.454**	**0.124**	**13.300**	0.000**	**0.635**	**0.498**	**0.811**
**Third quartile**	**1.248**	**0.401**	**9.703**	0.002**	**3.484**	**1.589**	**7.643**
**Upper adjacent**	**1.188**	**0.529**	**5.037**	0.025*	**3.281**	**1.162**	**9.259**
**SAM**	** − 0.448**	**0.194**	**5.314**	0.021*	**0.639**	**0.437**	**0.935**

*Abbreviations*: *SUV* standard uptake value, *TLG* total lesion glycolysis, *SAM* standardized added metabolic activity

*P< 0.05, **P< 0.01

### Performance of identification models

**Fig. 2:**
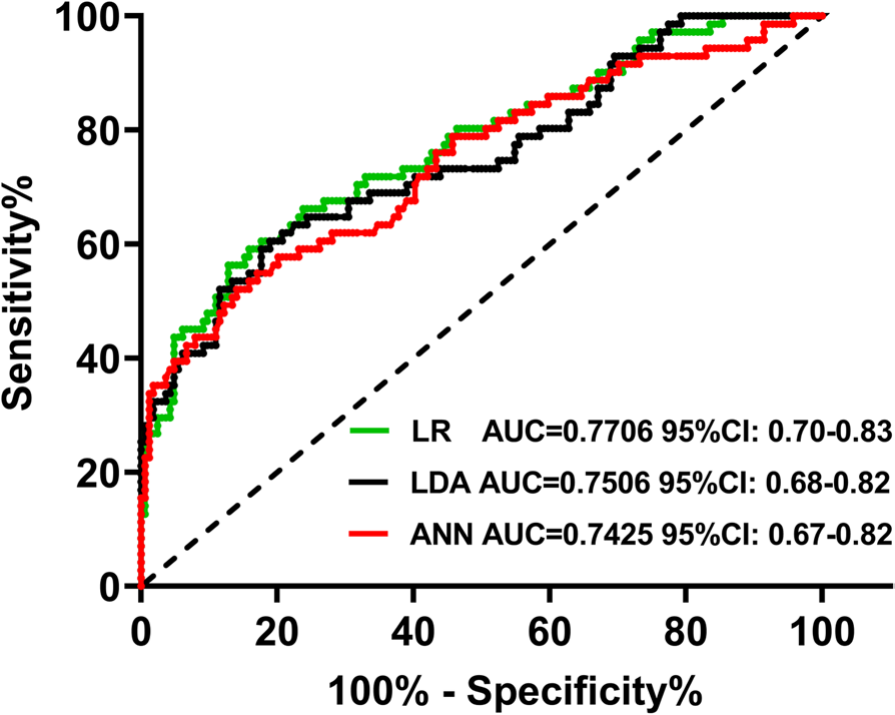
Receiver operating characteristic (ROC) curves of LR, LDA and ANN models. ROC curves show the performance of LR models (AUC = 0.7706; 95% CI 0.70–0.83) is better than LDA (AUC = 0.7506; 95% CI 0.68–0.82) and ANN (AUC = 0.7425; 95% CI 0.67–0.82) models in identifying the epileptogenic tubers of TSC patients

The PET quantitative indices from 235 tubers were used as the training set. The AUCs of the training set in the LR, LDA and ANN predictive models were 0.7706 (95% CI, 0.70–0.83), 0.7506 (95% CI, 0.68–0.82) and 0.7425 (95% CI, 0.67–0.82), respectively (Fig. 2), indicating that the LR model performed better in predicting epileptogenic tubers than the LDA and ANN models (Fig. 2). The LR model was internally evaluated based on ten-fold cross-validation on discrimination and calibration, and the results showed that it was well calibrated (Hosmer‒Lemeshow goodness-of-fit *p* value = 0.7). In addition, the multicollinearity of the PET quantitative indices was evaluated by the variance inflation factor (VIF), and the VIFs were less than 5, indicating that there was no multicollinearity in these models. Therefore, we selected the LR model to evaluate the values of the quantitative indices in predicting epileptogenic tubers.

**Fig. 3:**
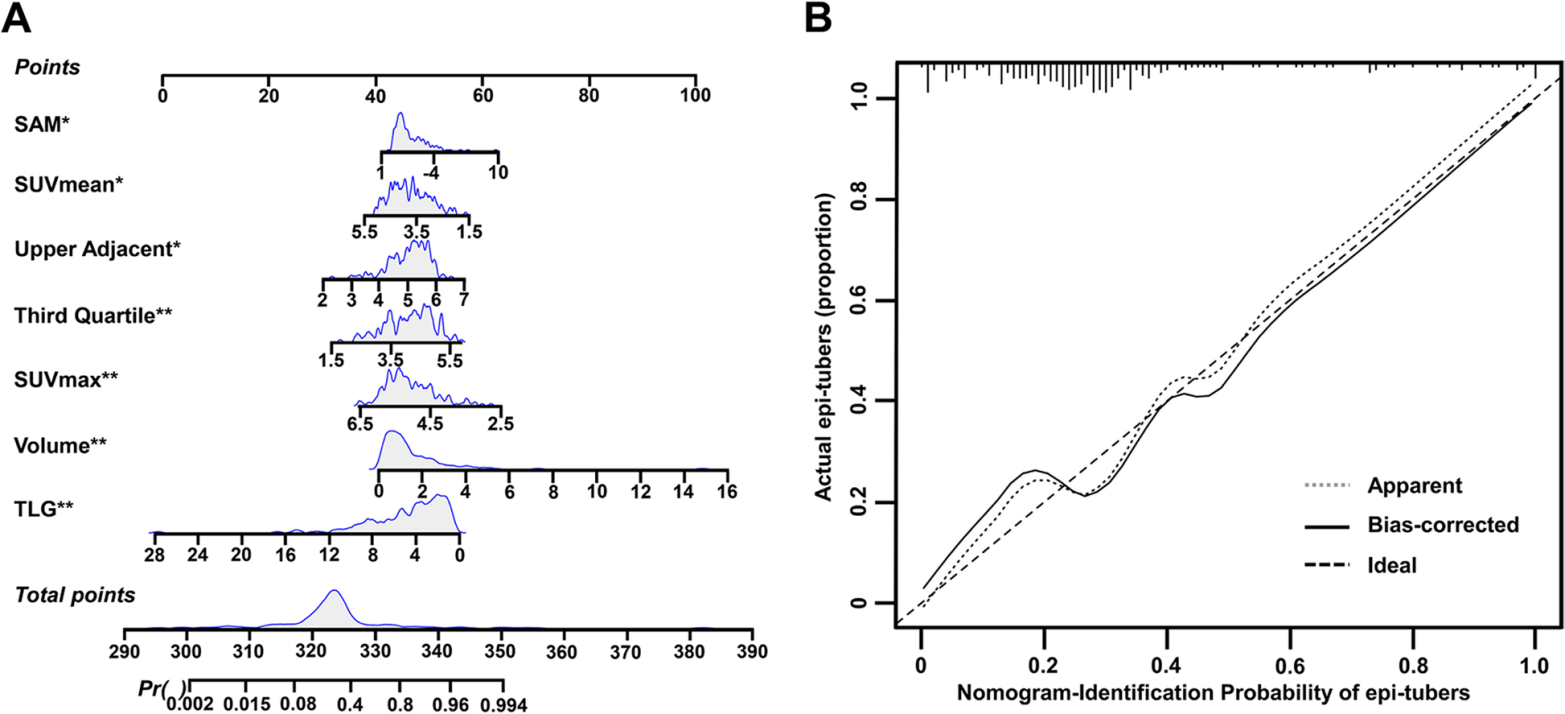
Discriminating nomogram.** A** Nomogram makes accessible for clinicians to identify the epileptogenic tubers according to quantitative indices of.^18^F-FDG PET images. Gray density plots describe the distribution of epileptogenic tubers in quantitative indices and total points. *P < 0.05, **P < 0.01. **B** Calibration curve for the nomogram to identify epileptogenic tubers. *X*-axis represents the nomogram-identification probability of epileptogenic tubers, and *Y*-axis represents the actual proportion of epileptogenic tubers. The diagonal dotted line indicates the best prediction by an ideal model. The apparent line represents the uncorrected performance of the nomogram, and the solid line shows the bias-corrected performance. 1000 bootstrap repetitions; mean absolute error = 0.046; *n* = 235

**Fig. 4:**
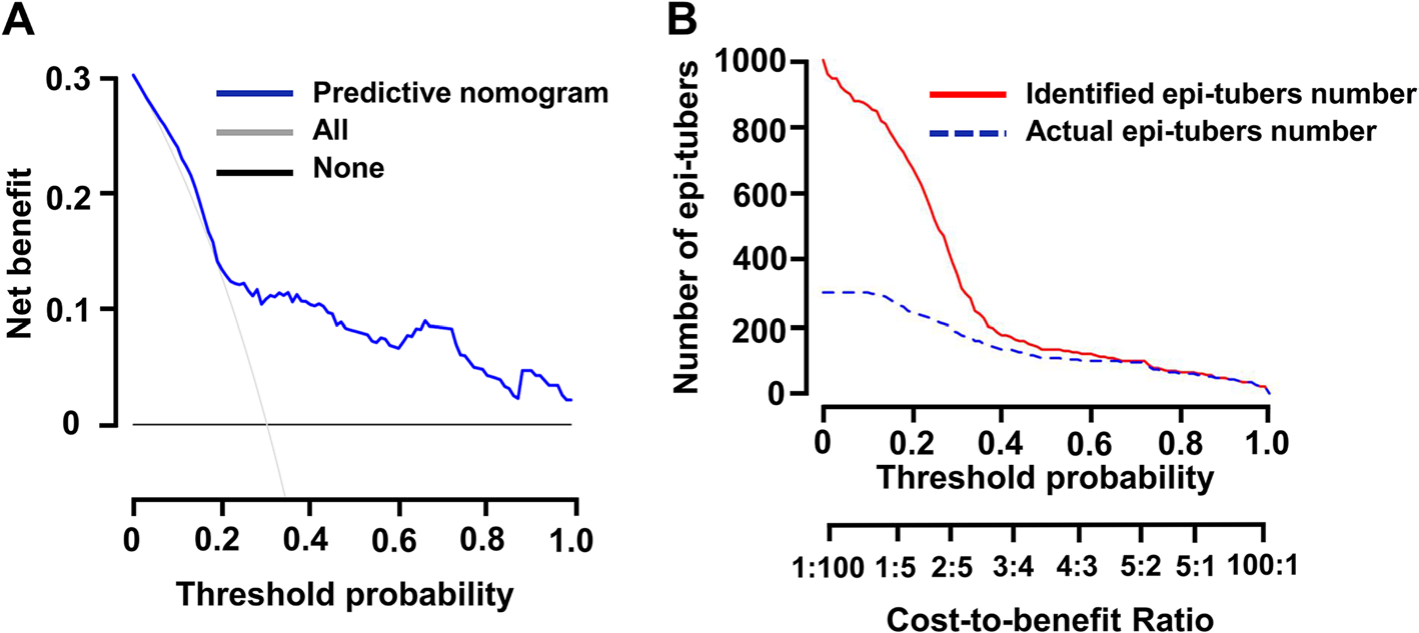
Decision curve and clinical impact curve show identification probability of epileptogenic tubers using nomogram.** A** Decision curve for the probability of identifying epileptogenic tubers using nomogram. The net benefits were measured at different threshold probabilities. The blue line represents the identification nomogram. The gray line represents the assumption that all tubers are identified as epileptogenic tubers. The black line represents the assumption that no tubers are identified as epileptogenic tubers. **B** Clinical impact curve to identify the epileptogenic tubers for a population size of 1000. The red curve shows the identified number of epileptogenic tubers at different threshold probabilities, and the blue curve represents actual number of epileptogenic tubers

Furthermore, a nomogram was constructed incorporating epileptogenic tuber-associated PET quantitative indices to guide clinicians in identifying epileptogenic tubers in TSC patients (Fig. 3A). Each patient was given points for each quantitative indices, and the higher the total points, the more likely the tuber was to be epileptogenic. In addition, calibration curves demonstrated that the nomogram had a similar performance to the ideal model and confirmed the good predictive capacity of the nomogram (Fig. 3B). DCA (Fig. 4A) and CIC (Fig. 4B) were established to confirm the clinical applicability of the nomogram. DCA showed that the prediction of epileptogenic tubers with the nomogram provide greater net benefits when the high-risk threshold probability was > 30%. CIC was used to predict the stratification of the probability of epileptogenic tubers for a sample size of 1000 and suggested that the number of identified epileptogenic tubers was close to the actual number of epileptogenic tubers when the threshold probability was higher than 0.4, and the cost-to-benefit ratio was 0.75.

### Evaluation of the prediction model

New PET data of 32 tubers from 7 patients were used as the testing set to evaluate the predictability of the LR model. The AUC of the LR model was 0.8502 (95% CI, 0.71–0.99), and the accuracy was 78.13% (Table 3). Furthermore, we evaluated the prognostic value of the LR model in the long-term outcomes of cortical tubers based on the 1-, 3- and 5-year follow-up data. In the 1-year follow-up of 74 epileptogenic tubers, the AUC value was 0.7805 (95% CI, 0.71–0.85), and the accuracy was 79.15%. In the 3-year follow-up of 77 epileptogenic tubers, the AUC value was 0.8066 (95% CI, 0.74–0.87), and the accuracy was 80.43%. In the 5-year follow-up of 80 epileptogenic tubers, the AUC value reached 0.8172 (95% CI, 0.75–0.87), and the accuracy was 79.15% (Table 3). These findings showed the capability of the LR model to predict the long-term outcomes of cortical tubers.

**Table 3:** Identification of LR model to epileptogenic tubers in TSC patients

Characteristics	AUC	AUC 95%CI	Accuracy
Testing sets	**0.8502**	**0.71–0.99**	**0.7813**
1 year follow	**0.7805**	**0.71–0.85**	**0.7915**
3 years follow	**0.8066**	**0.74–0.87**	**0.8043**
5 years follow	**0.8172**	**0.75–0.87**	**0.7915**

*Abbreviations*: *LR* logistic regression, *TSC* tuberous sclerosis complex, *AUC* area under curve

## Discussion

A quantitative predictive model for preoperatively identifying epileptogenic tubers in TSC patients based on ^18^F-FDG PET images is lacking. In this study, LR, LDA and ANN models were established based on the quantitative indices derived from ^18^F-FDG PET images and effectively identified epileptogenic tubers from all cortical tubers. ROC analysis showed that the LR model performed better in predicting epileptogenic tubers than the LDA and ANN models. A practical nomogram was constructed and showed good clinical applicability in identifying epileptogenic tubers according to DCA and CIC. Intriguingly, the LR model could also predict the long-term outcomes of cortical tubers. This study provided a noninvasive method for identifying epileptogenic tubers and predicting long-term outcomes of cortical tubers in patients with TSC.

Over the past three decades, ^18^F-FDG PET has been used to map patterns of brain glucose metabolism to evaluate brain function or demonstrate metabolism abnormalities in various brain disorders [26]. It is a promising modality with high sensitivity, specificity and accuracy in detecting foci in patients with neurological diseases and is thought to have higher sensitivity than MRI [12]. Moreover, ^18^F-FDG PET has been widely used in the presurgical assessment of patients with DRE by identifying the hypometabolic seizure onset zones, especially when MRI and scalp EEG are unable to do so or are discordant [11]. Previous studies have shown that interhemispheric metabolic asymmetry on interictal PET could indicate the location of the epileptogenic zone, and the oxygen-glucose index based on PET images showed unique value in identifying foci during the interictal period in patients with DRE [27, 28]. PET serves an important role in understanding the neuro‑behavioural characteristics of TSC patients, including autism, attention deficit hyperactivity disorder and cognitive impairment, and it has become an indispensable, noninvasive clinical approach for preoperative evaluation in TSC patients with intractable epilepsy [29–32]. Notably, lower values of the PET quantitative indices SUV and TLG have been identified in lesions of FCDII patients than in contralateral and control regions, and ipsilateral relative SUVs were associated with increased lateralized periodic discharge frequency [16, 33]. Furthermore, our previous study confirmed that the SUVmax and SUVmean were significantly decreased in epileptogenic tubers [17]. In the current study, epileptogenic tubers had lower values for the quantitative indices SUVmax, SUVmean and TLG and a greater volume than non-epileptogenic tubers. Intriguingly, these quantitative indices were negatively correlated with seizure frequency, seizure duration and typical hyperactivation of the mTOR signalling pathway in TSC patients. Based on these results, we hypothesized that these PET quantitative indices had potential value in identifying epileptogenic tubers.

Further, LR, LDA and ANN predictive models were constructed based on the PET quantitative indices, and ROC analysis showed that the LR model had greater predictive value in identifying epileptogenic tubers than the LDA and ANN models. LR, a sensitive and stable binary prediction machine learning method, is widely used in feature-based classification, and it has been applied to single-neuron recordings from depth-electrode microwires to predict seizure onset zones [34, 35]. In addition, a predictive nomogram was constructed to make accurate localization assessments to quantitatively predict epileptogenic tubers in a personalized way. DCA is an appropriate method to evaluate alternative diagnosis and prognostic strategies by analyzing the clinical net benefit with a probability threshold and has previously been used for the withdrawal of antiseizure medications [36]. A previous study reported that DCA confirmed the usefulness and benefits of a clinical model for predicting seizure freedom in MRI-negative epilepsy patients treated with antiseizure medications [37]. The DCA results further provided evidence of the usefulness and benefits of our predictive model.

Previous studies have shown that α-[(11)C]-methyl-L-tryptophan (AMT)-PET could pinpoint hypermetabolic epileptogenic tubers in approximately two-thirds of patients with tuberous sclerosis and intractable epilepsy, provide independent complementary information on the localization of epileptogenic regions in TSC and enhance the confidence of successful epilepsy surgery [38–40]. There are some limitations to the clinical promotion and application of AMT PET, including the very short half-life of its tracer and its low sensitivity in localizing epileptogenic tubers [41]. In contrast, ^18^F-FDG PET is commonly used in clinical practice, but few studies have used it to identify epileptogenic tubers. We constructed the LR predictive model based on the quantitative indices of ^18^F-FDG PET, achieving an AUC of 0.7706. New PET images from cortical tubers were used as a testing set to evaluate the predictive capability of the LR model; the AUC was 0.8502, and the accuracy was 78.13%. These results indicated that the ability of ^18^F-FDG PET to identify epileptogenic tubers is comparable to that of AMT PET.

Some non-epileptogenic cortical tubers can potentially develop into epileptogenic tubers, but few studies have focused on the true outcomes of cortical tubers [4]. Thus, we explored the predictability of the LR model regarding the outcomes of cortical tubers using the follow-up data. Interestingly, the AUC and accuracy of the LR model improved with greater follow-up times, suggesting that the LR model could predict the outcomes of epileptogenic tubers and guide clinical intervention in TSC patients.

## Conclusions

In conclusion, we developed a predictive LR model to identify epileptogenic tubers in TSC patients based on ^18^F-FDG PET quantitative indices. Remarkably, the LR model also exhibited a promising ability to predict the longer-term outcomes of cortical tubers. The noninvasive predictive model can provide guidance for neurosurgical intervention in TSC, but further studies are required to confirm our findings in clinical practice.

### Supplementary Information

**Additional file 1: Table S1.** The detailed explanation of Quantitative Indices. **Table S2.** ^18^F-FDG PET Quantitative Indices of Epileptogenic and Non-Epileptogenic tubers in TSC patients. **Figure S1.** Illustration shows the image processing pipeline and quantitative indices analysis based on ^18^F-FDG PET images. **Figure S2.** Relationship between the PET quantitative indices (SUVmean, SUVmax, Volume, TLG) and epileptic characteristics in TSC patients with single epileptogenic tuber. **Figure S3.** Relationship between the PET quantitative indices and mTOR expression in epileptogenic tubers.

**Additional file 2: Figure S1.** The original blot images of mTOR and GAPDH in Additional file 1 Fig. S3F.

## Data Availability

The datasets used and/or analysed during the current study are available from the corresponding author on reasonable request.

## Abbreviations

ANN: Artificial neural network
ASM: Antiseizure medication
AUC: Area under curve
CIC: Clinical impact curve
CT: Computed tomography
DCA: Decision curve analysis
DRE: Drug-resistant epilepsy
LDA: Linear discriminant analysis
LR: Logistic regression
MRI: Magnetic resonance imaging
PET: Positron emission tomography
RMSD: Root-mean-squared deviations
ROC: Receiver operating characteristic curve
ROI: Regions of interests
SAM: Standardized added metabolic
SEEG: Stereoelectroencephalogram
SUV: Standard uptake value
TLG: Total lesion glycolysis
TLG: Total lesion glycolysis
TSC: Tuberous sclerosis complex
