# Supplementary material for: Predictive model for epileptogenic tubers from all tubers in patients with tuberous sclerosis complex based on 18F-FDG PET: an 8-year single-centre study
Source: BMC Med. 2023 Dec 18;21:500. doi: 10.1186/s12916-023-03121-0 (PMC10729377; doi:10.1186/s12916-023-03121-0)
Supplement: Supplementary file 1 — Additional file 1: Table S1. The detailed explanation of Quantitative Indices. Table S2. 18F-FDG PET Quantitative Indices of Epileptogenic and Non-Epileptogenic tubers in TSC patients. Figure S1. Illustration shows the image processing pipeline and quantitative indices analysis based on 18F-FDG PET images. Figure S2. Relationship between the PET quantitative indices (SUVmean, SUVmax, Volume, TLG) and epileptic characteristics in TSC patients with single epileptogenic tuber. Figure S3. Relationship between the PET quantitative indices and mTOR expression in epileptogenic tubers. [file 12916_2023_3121_MOESM1_ESM.doc]

**Additional file 1 for**

**Predictive model for epileptogenic tubers from all tubers in patients with tuberous sclerosis complex based on 18F-FDG PET: an 8-year single-centre study**

Zhongke Wang^1*^, Yang Li^2*^, Zeng He^2*^, Shujing Li^2*^, Kaixuan Huang^2^, Xianjun Shi^2^, Xiaoqin Sun^2^, Ruotong Ruan^3^, Chun Cui^4^, Ruodan Wang^5^, Li Wang^5^, Shengqing Lv^2^, Chunqing Zhang^2,6^, Zhonghong Liu^1^, Hui Yang^2,6#^, Xiaolin Yang^2#^, Shiyong Liu^2,6#^

**Corresponding authors:**

Shiyong Liu ([liushi24252016@163.com](mailto:liushi24252016@163.com)), Xiaolin Yang ([yangxl2013@163.com](mailto:yangxl2013@163.com)) and Hui Yang ([13808390069@163.com](mailto:13808390069@163.com))

**Additional file 1: Table S1-S2, Figure S1-S3.**

| **Table S1** The detailed explanation of Quantitative Indices.   | **No** | **Abbreviation** | **Full name** | | --- | --- | --- | | **1** | **SUVmean** | mean value in ROI (g/ml) | | **2** | **SUVmax** | maximum value in ROI (g/ml) | | **3** | **SUVmin** | minimum value in ROI (g/ml) | | **4** | **RMSD** | root-mean-square deviations in ROI (g/ml) | | **5** | **Volume** | volume of ROI (cm^3) | | **6** | **First Quartile** | 25th percentile value in ROI (g/ml) | | **7** | **Third Quartile** | 75th percentile value in ROI (g/ml) | | **8** | **Upper Adjacent** | first value in ROI not greater than 1.5 times the interquartile range (g/ml) | | **9** | **TLG** | total lesion glycolysis (g) | | **10** | **Glycolysis Q1** | lesion glycolysis calculated from the first quarter of the grayscale range within the ROI (g) | | **11** | **Glycolysis Q2** | lesion glycolysis calculated from the second quarter of the grayscale range within the ROI (g) | | **12** | **Glycolysis Q3** | lesion glycolysis calculated from the third quarter of the grayscale range within the ROI (g) | | **13** | **Glycolysis Q4** | lesion glycolysis calculated from the fourth quarter of the grayscale range within the ROI (g) | | **14** | **Q1 Distibution** | percent of gray values that fall within the first quarter of the grayscale range within the ROI (%) | | **15** | **Q2 Distibution** | percent of gray values that fall within the second quarter of the grayscale range within the ROI (%) | | **16** | **Q3 Distibution** | percent of gray values that fall within the third quarter of the grayscale range within the ROI (%) | | **17** | **Q4 Distibution** | percent of gray values that fall within the fourth quarter of the grayscale range within the ROI (%) | | **18** | **SAM** | standardized added metabolic activity (g) | | **19** | **SAM mean background** | intermediate value for calculating SAM, estimates local background near ROI (g/ml) |   **Table S2** ^18^F-FDG PET Quantitative Indices of Epileptogenic and Non-Epileptogenic tubers in TSC patients. | | | |  |
| --- | --- | --- | --- | --- | --- | --- | --- | --- | --- | --- | --- | --- | --- | --- | --- | --- | --- | --- | --- | --- | --- | --- | --- | --- | --- | --- | --- | --- | --- | --- | --- | --- | --- | --- | --- | --- | --- | --- | --- | --- | --- | --- | --- | --- | --- | --- | --- | --- | --- | --- | --- | --- | --- | --- | --- | --- | --- | --- | --- | --- | --- | --- | --- | --- |
|  |
| **Quantitative Indices** | **Epileptogenic tubers (n=71)** | **Non-Epileptogenic tubers (n=164)** | ***P* Value** |  |
| **SUVmean** | **3.59 ± 0.67** | **3.86 ± 0.72** | ***P* = 0.008**** |  |
| **SUVmax** | **4.90 ± 0.74** | **5.20 ± 0.63** | ***P* = 0.002**** |  |
| **SUVmin** | **2.22 ± 0.75** | **2.40 ± 0.89** | ***P* = 0.135** |  |
| **RMSD** | **3.67 ± 0.66** | **3.92 ± 0.70** | ***P* = 0.011*** |  |
| **Volume** | **1.74 ± 1.91** | **1.32 ± 1.15** | ***P* = 0.043*** |  |
| **First Quartile** | **3.20 ± 0.86** | **3.17 ± 0.83** | ***P* = 0.782** |  |
| **Third Quartile** | **4.25 ± 0.83** | **4.07 ± 0.75** | ***P* = 0.103** |  |
| **Upper Adjacent** | **5.03 ± 0.65** | **5.06 ± 0.68** | ***P* = 0.755** |  |
| **TLG** | **3.89 ± 2.34** | **4.95 ± 3.84** | ***P* = 0.032*** |  |
| **Glycolysis** |  |  |  |  |
| **Q1** | **0.60 ± 0.41** | **0.64 ± 0.51** | ***P* = 0.559** |  |
| **Q2** | **1.97 ± 1.25** | **2.01 ± 1.67** | ***P* = 0.839** |  |
| **Q3** | **2.27 ± 1.61** | **2.40 ± 2.07** | ***P* = 0.658** |  |
| **Q4** | **0.78 ± 0.61** | **0.81 ± 0.71** | ***P* = 0.749** |  |
| **Distribution** |  |  |  |  |
| **Q1** | **20.96 ± 9.19** | **21.49 ± 8.98** | ***P* = 0.679** |  |
| **Q2** | **35.47 ± 10.75** | **35.77 ± 9.57** | ***P* = 0.829** |  |
| **Q3** | **30.95 ± 9.41** | **31.16 ± 10.21** | ***P* = 0.880** |  |
| **Q4** | **11.76 ± 6.73** | **13.27 ± 7.80** | ***P* = 0.159** |  |
| **SAM** | **-1.84 ± 1.30** | **-1.62 ± 1.40** | ***P* = 0.266** |  |
| **SAM mean background** | **5.04 ± 1.16** | **5.14 ± 1.09** | ***P* = 0.524** |  |
| Abbreviations: SUV, standard uptake value; RMSD, root-mean-squared deviations; TLG, total lesion glycolysis; SAM, standardized added metabolic activity. Values are mean ± SD. | | | |  |


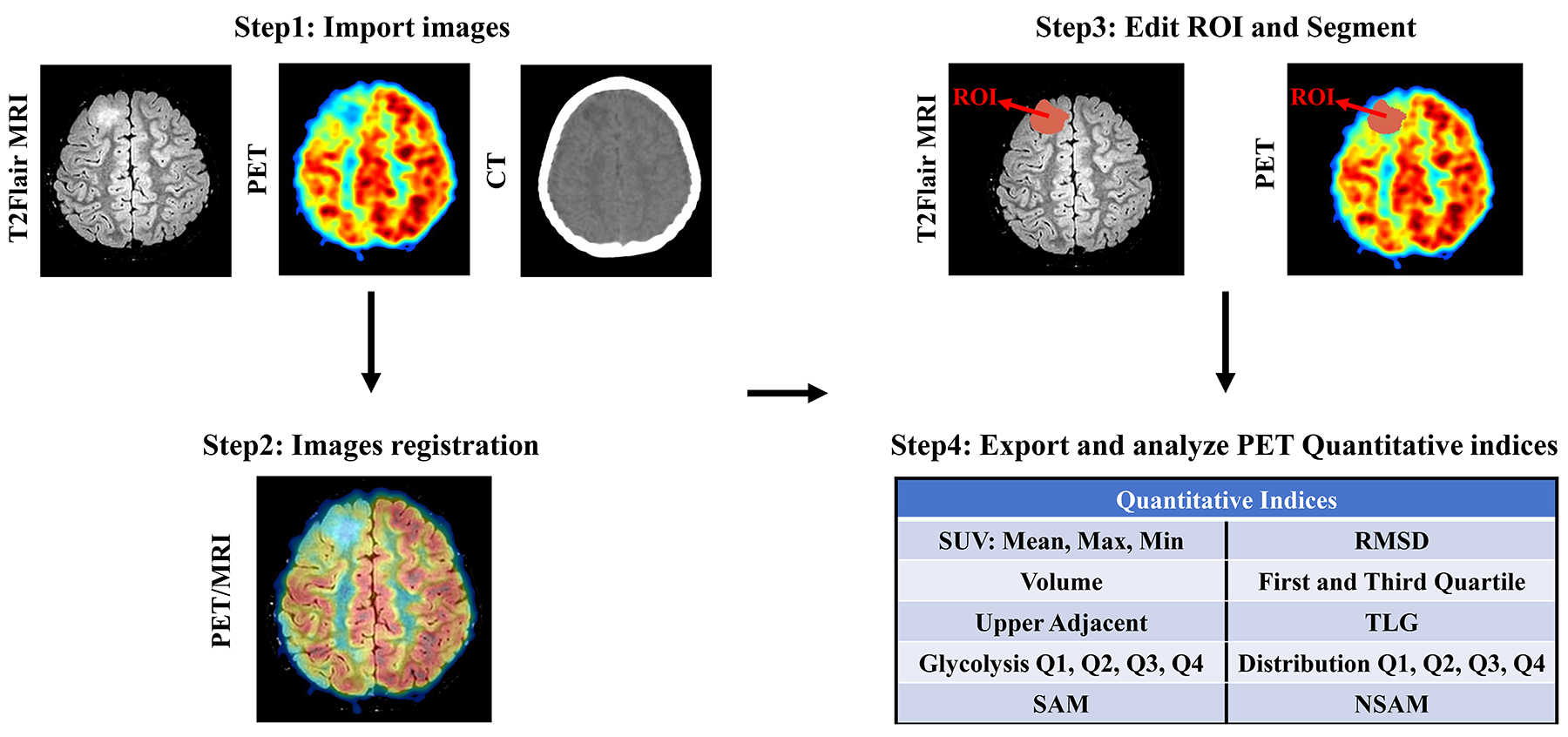
**Figure S1**

**Figure S1 Illustration shows the image processing pipeline and quantitative indices analysis based on ^18^F-FDG PET images.** 3DSlicer and R 4.2.1 software were used in this process. Red arrows indicate the ROIs.


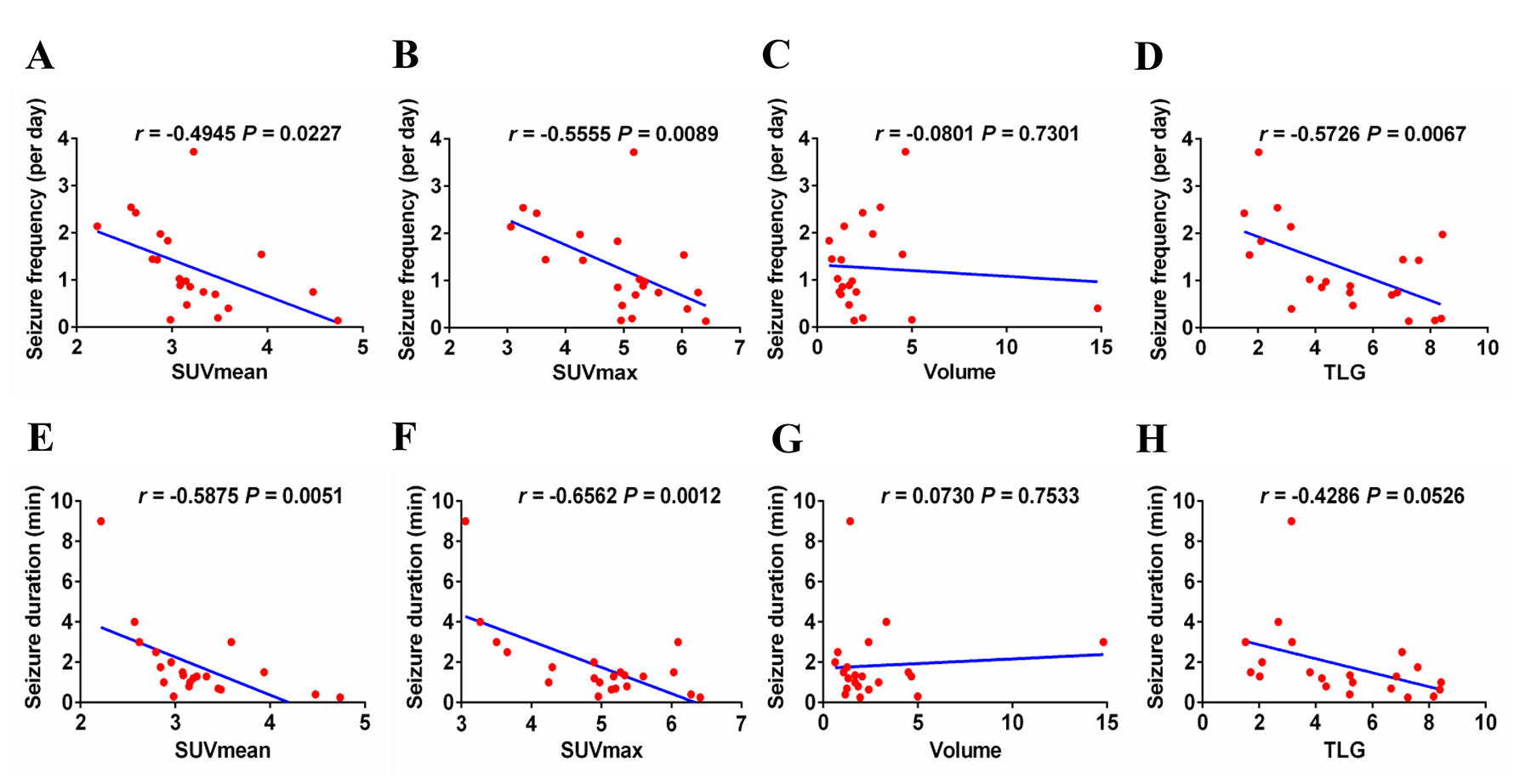
**Figure S2**

**Figure S2:** **Relationship between the PET quantitative indices (SUVmean, SUVmax, Volume, TLG) and epileptic characteristics in TSC patients with single epileptogenic tuber.** (A, E) Negative association of seizure frequency (r = -0.4945, *P* = 0.0227) and duration (r = -0.5875, *P* = 0.0051) with SUVmean. (B, F) Negative association of seizure frequency (r = -0.5555, *P* = 0.0089) and duration (r = -0.6562, *P* = 0.0012) with SUVmax. (C, G) No association of seizure frequency and duration with Volume. (D, H) Negative association of seizure frequency (r = -0.5726, *P* = 0.0067) and duration (r = -0.4286, *P* = 0.0526) with TLG.


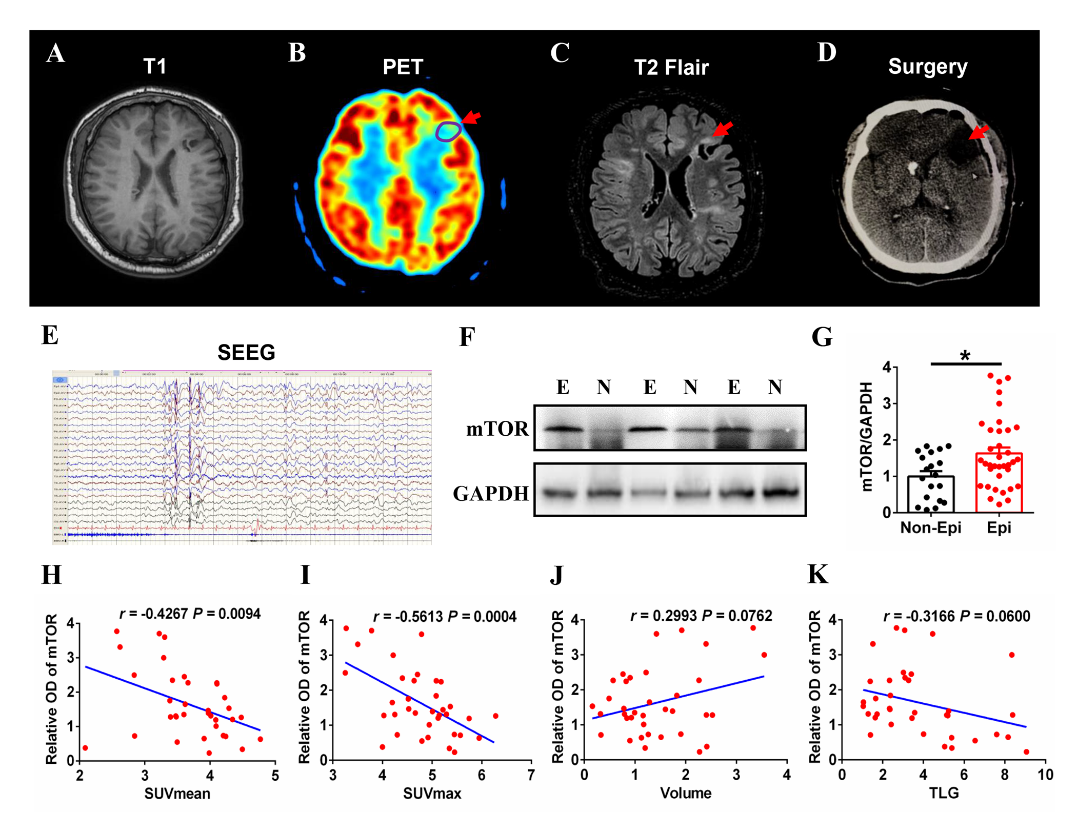
**Figure S3**

**Figure S3: Relationship between the PET quantitative indices and mTOR expression in epileptogenic tubers.** (A-E) Preoperative imaging examination and EEG monitoring. (F-G) Increased mTOR expression in epileptogenic tubers (n = 36) than non-epileptogenic tubers (n = 19). (H) Negative association of mTOR expression (r = -0.4267, *P* = 0.0094) with SUVmean. (I) Negative association of mTOR expression (r = -0.5613, *P* = 0.0004) with SUVmax. (J-K) No association of mTOR expression with Volume and TLG. Red arrows indicate the epileptogenic tuber.
