# Supplementary material for: Predictive model for epileptogenic tubers from all tubers in patients with tuberous sclerosis complex based on 18F-FDG PET: an 8-year single-centre study
Source: BMC Med. 2023 Dec 18;21:500. doi: 10.1186/s12916-023-03121-0 (PMC10729377; doi:10.1186/s12916-023-03121-0)
Supplement: Supplementary file 2 — Additional file 2: Figure S1. The original blot images of mTOR and GAPDH in Additional file 1 Fig. S3F. [file 12916_2023_3121_MOESM2_ESM.doc]

**Additional file 2: Figure S1.**


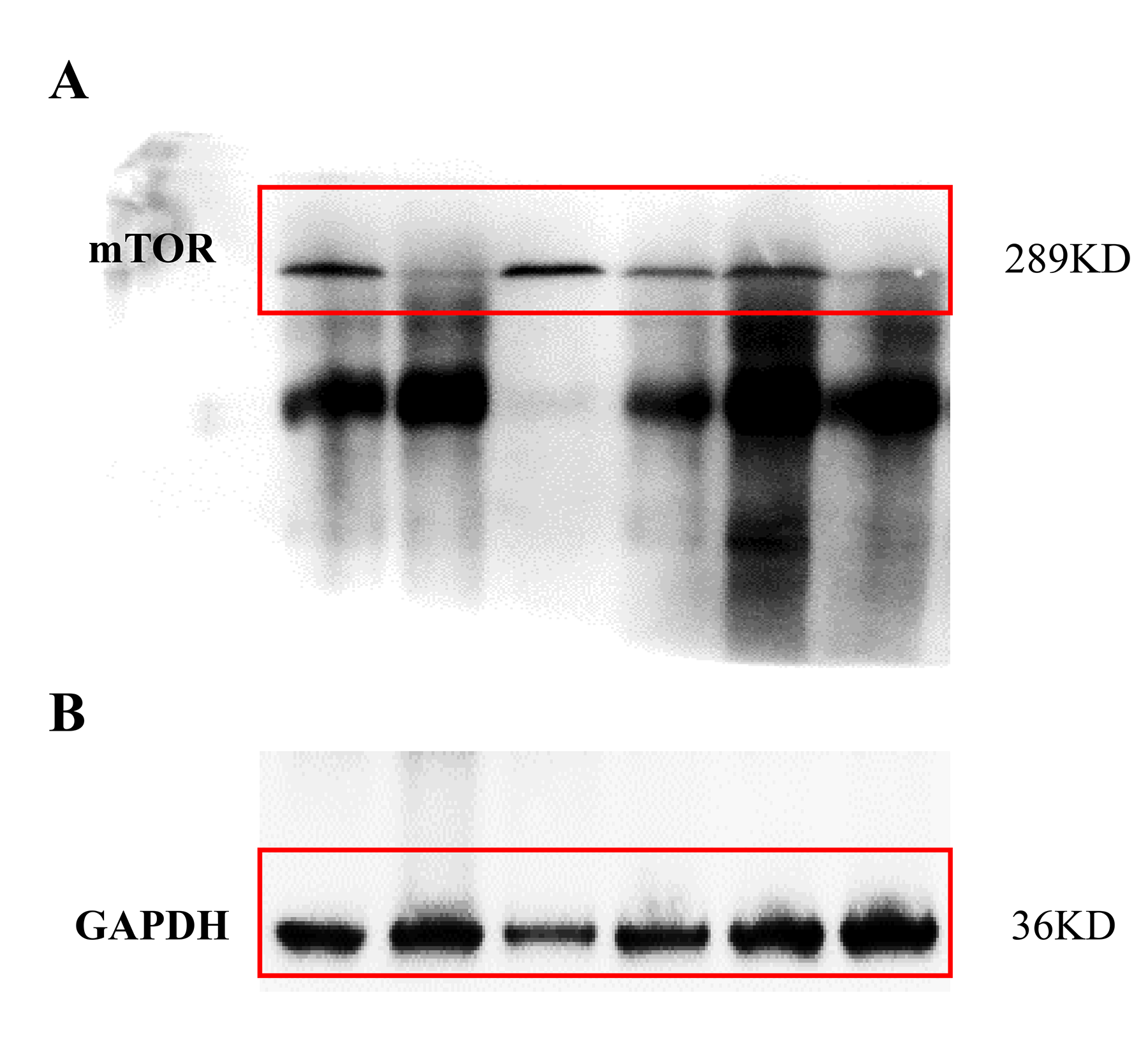
**Figure S1**

**Figure S1:** **The original blot images of mTOR and GAPDH in Additional file 1: Fig. S3F.** (A) Original blot image of mTOR. (B) Original blot image of GAPDH.
